# UK ambulance service resuscitation management of pulseless electrical activity: a systematic review protocol of text and opinion

**DOI:** 10.29045/14784726.2020.06.5.1.20

**Published:** 2020-06-01

**Authors:** Alison Coppola, Sarah Black, Sasha Johnston, Ruth Endacott

**Affiliations:** The University of Plymouth; South Western Ambulance Service NHS Foundation Trust; South Western Ambulance Service NHS Foundation Trust; The University of Plymouth; South Western Ambulance Service NHS Foundation Trust; The University of Plymouth

**Keywords:** out-of-hospital cardiac arrest, paramedics, pre-hospital resuscitation, pulseless electrical activity, termination of resuscitation, UK ambulance services

## Abstract

**Background:**

Out-of-hospital cardiac arrest patients with pulseless electrical activity are treated by paramedics using basic and advanced life support resuscitation. When resuscitation fails to achieve return of spontaneous circulation, there are limited evidence and national guidelines on when to continue or stop resuscitation. This has led to ambulance services in the United Kingdom developing local guidelines to support paramedics in the resuscitative management of pulseless electrical activity. The content of each guideline is unknown, as is any association between guideline implementation and patient survival. We aim to identify and synthesise local ambulance service guidelines to help improve the consistency of paramedic-led decision-making for the resuscitation of pulseless electrical activity in out-of-hospital cardiac arrest.

**Methods:**

A systematic review of text and opinion will be conducted on ambulance service guidelines for resuscitating adult cardiac arrest patients with pulseless electrical activity. Data will be gathered direct from the ambulance service website. The review will be guided by the methods of the Joanna Briggs Institute (JBI). The search strategy will be conducted in three stages: 1) a website search of the 14 ambulance services; 2) a search of the evidence listed in support of the guideline; and 3) an examination of the reference list of documents found in the first and second stages and reported using the Preferred Reporting Items for Systematic Reviews and Meta-analyses. Each document will be assessed against the inclusion criteria, and quality of evidence will be assessed using the JBI Critical Appraisal Checklist for Text and Opinion. Data will be extracted using the JBI methods of textual data extraction and a three-stage data synthesis process: 1) extraction of opinion statements; 2) categorisation of statements according to similarity of meaning; and 3) meta-synthesis of statements to create a new collection of findings. Confidence of findings will be assessed using the graded ConQual approach.

## Introduction

In the United Kingdom (UK), ambulance services resuscitate over 28,000 adults each year following cardiac arrest (Hawkes et al., 2017), of which approximately 31% present with the non-shockable rhythm pulseless electrical activity (OHCAO Project Team, 2017). UK paramedics treat PEA using basic and advanced life support which focuses on reversing the treatable causes of cardiac arrest (Myerburg et al., 2013). When a reversible cause is not found, patient survival is unlikely, with a reported UK survival-to-discharge rate of 4.5% (OHCAO Project Team, 2017). Despite the low rate of survival, when compared to shockable rhythms, there is limited evidence about when to terminate resuscitation for PEA. This may explain why some patients are conveyed to hospital with ongoing resuscitation, even when it is considered futile (House et al., 2017).

A number of studies have attempted to validate termination of resuscitation rules. However, these studies have reported conflicting results due to a difference in local strategy and the small number of patients who survived when the termination of resuscitation criteria were met (Verhaert et al., 2016). As a result, there is no national guidance on when resuscitation can be discontinued in PEA, and UK ambulance services have had to develop their own local guidelines to assist their staff in these circumstances (House et al., 2017). However, the local guideline of each ambulance service is unknown, as is the impact of each guideline on patient outcomes.

This review aims to explore and evaluate local clinical guidelines for terminating resuscitation in PEA. However, given the lack of high-quality evidence, it is necessary to utilise evidence derived from clinical expertise and opinion. Therefore, this systematic review will comprise text and opinion (McArthur et al., 2015).

## Objectives

The objectives of this review are to:

- summarise the current variation in treatments
- summarise the evidence cited in support of such treatments

To develop the review protocol, a question was formed using the population, phenomenon of interest and context outcome criteria (PPC) (Sackett et al., 1996). The population for the review is adult PEA; the phenomenon of interest is the local resuscitative management and the context is UK ambulance services. The question is: What are the resuscitation guidelines and supporting evidence for cardiac arrest patients with PEA treated by UK ambulance services?

## Methods

This review will be guided by the methods of the Joanna Briggs Institute (JBI) (McArthur et al., 2017). JBI have formed a robust methodology to include search strategies, critical appraisal, data extraction and quality assessment to ensure reviews have a valid role within healthcare.

### Initial search

An initial Google search for local guidelines was conducted, and two relevant documents were found: the resuscitation policies for the Yorkshire Ambulance NHS Trust (Millins et al., 2018) and the South Central Ambulance Trust (Sherwood, 2018). Each guideline provided a different strategy for managing resuscitation for PEA. A preliminary search of systematic reviews from the JBI database, Cochrane Review Library, Prospero and NIHR database for reviews identified no registered protocols or systematic reviews for this topic area.

### Eligibility criteria

#### Population of interest

This review will consider the local clinical guidelines that manage patients over 18 years old. The population of interest will have suffered a pre-hospital cardiac arrest and present with the non-shockable rhythm PEA.

#### Phenomena of interest

This review will consider local pre-hospital clinical guidelines and cited evidence within, which underpin the resuscitative management of PEA cardiac arrest. Local clinical guidelines are of interest as there is a paucity of national clinical guidance or consensus surrounding the topic area.

### Context

This review will consider local clinical guidelines from the 14 ambulance services in the UK. The geographical location will capture local-level guidelines, which will contribute to broadening the evidence base and informing future steps towards a national perspective.

### Information sources

This review is concerned with local clinical guidelines and the evidence cited in support of them. Only UK clinical guidelines will be considered, as the emergency medical systems in other countries differ from physician to community-led resources and are therefore not comparable to UK-based practice (Ong et al., 2012).

Local clinical guidelines published from 2015 will be considered. These time parameters reflect the most recent published guidelines from the UK Resuscitation Council and Joint Royal College Ambulance Liaison Committee. If a guideline is found to precede 2015 or is not available via the ambulance service website, a written request will be made to the ambulance service, to ensure the most up-to-date guideline is included. Clinical guidelines that consist of qualitative and quantitative evidence will be considered for synthesis, as often complex health interventions consist of both methodologies (Glenton et al., 2014).

### Search strategy

The search strategy will focus on local clinical guidelines and the evidence cited in support of the guideline. It is possible that local guidelines may draw upon a range of sources, including expert opinion, national guidelines and published research. Therefore, reference searches will encompass text, publications and research studies (McArthur et al., 2017). In addition, a search for unpublished literature will be conducted to reduce publication bias and ensure the best available data is found (Faggion et al., 2016). The search will be undertaken in three stages.

The first stage will focus on the UK ambulance services as listed below:

- East Midlands Ambulance Service NHS Trust
- East of England Ambulance Service NHS Trust
- Isle of Wight NHS Trust
- London Ambulance Service NHS Trust
- North East Ambulance Service NHS Trust
- North West Ambulance Service NHS Trust
- South Central Ambulance Service NHS Foundation Trust
- South East Coast Ambulance Service NHS Foundation Trust
- South Western Ambulance Service NHS Foundation Trust
- West Midlands Ambulance Service NHS Trust
- Yorkshire Ambulance Service NHS Trust
- Northern Ireland Ambulance Service
- Scottish Ambulance Service
- Welsh Ambulance Service

Ambulance service websites will be searched. Where local guidelines are unavailable or not found, a written request for the guideline will be made to the National Ambulance Research Steering Group and the National Ambulance Lead Paramedic Group. The use of unpublished literature has caused concern due to the uncontrollable amount and lack of quality assessment (Adams et al., 2017). With this in mind, a JBI review provides the opportunity to critically appraise and assess the authenticity of the evidence to ensure a valid and robust review (McArthur et al., 2017).

The second stage aims to identify the evidence listed in support of the local guideline. The third stage will examine the reference list of documents found in the first and second stages of the search. This hand search will reduce publication bias by ensuring all documents found are included in the review (Vassar et al., 2016).

### Data management

The results of the search will be collected and uploaded to the reference manager Mendeley 1.19.3 (https://www.mendeley.com). Duplicates will be removed. The selected documents will be extracted and uploaded to the System for the Unified Management, Assessment and Review of Information from JBI (Munn et al., 2018).

To standardise and report the search results, the Preferred Reporting Items for Systematic Reviews and Meta-analyses (PRISMA) will be applied (Moher et al., 2010).

### Selection process

The documents will be screened against the eligibility criteria. To increase the credibility of this review, each document will be screened by two independent reviewers. Disagreement will be resolved by discussion or by introducing a third reviewer (McArthur et al., 2017). The rationale for excluding documents will be reported and filed in the appendices to ensure the results are transparent and auditable (McDonagh et al., 2013).

### Data collection process

All documents will be subjected to critical appraisal and include a focus on genuine opinion, driving motivation, the location of the documents, and related conflict to ensure transparency (McArthur et al., 2017). This approach aims to reduce the risk of bias for selected documents (McDonagh et al., 2013). Documents will be assessed using two reviewers, and disagreements surrounding quality will be addressed by a third reviewer. The results from the critical appraisal are presented in Table 1.

**Table 1.** JBI Critical Appraisal Checklist for Text and Opinion (McArthur et al., 2017).

Is the opinion source clearly identified?	Does the source of opinion have standing in the field of expertise?	Are the interests of the relevant population the central focus of the opinion?	Is the stated position the result of an analytical process, and is there logic in the opinion expressed?	Is there reference to the extant literature?	Is any incongruence with the literature/sources logically defended?

### Data items

The JBI SUMARI extraction tool will be used to transfer the main conclusions found within the text (McArthur et al., 2017). Data extraction will be conducted using two reviewers. Any disagreements between reviewers will be resolved by introducing a third (Mathes et al., 2017). Aligned with JBI methods for textual data extraction, the following table headings will be applied:

- Ambulance Trust (context)
- Year of publication
- Review date
- Type of text
- Population presented
- The panel who developed the guideline
- The sources cited to inform the guideline
- Conclusions relevant to the objectives of this review
- Reviewer notes

For evidence cited in support of the local guidelines, the data extraction table headings will include:

- Type of text
- Those represented
- Setting
- Geographical content
- Cultural content
- Logic of argument
- Conclusion
- Reviewer comments

The table format aims to reduce error and to record an accurate account of categorisation and synthesis (McArthur et al., 2017). This format will ensure that the extraction tool accounts for the different types of evidence found (Munn et al., 2018).

## Outcomes

Each document will be carefully read to identify accurate statements which encompass the main conclusions. The data will be extracted consistently and standardised by using the extraction tool to meet the main outcomes of this review:

- Identify the local strategies for terminating resuscitation for cardiac arrest patients with PEA for each ambulance service
  - Identify when to continue or cease resuscitation
  - Identify any other related guidance
- Identify the evidence cited in support of such treatments

### Risk of bias and quality assessment

The quality of evidence will be assessed using the Joanna Briggs Institute Critical Appraisal Checklist for Text and Opinion (McArthur et al., 2017). The appraisal checklist is an important tool to evaluate the quality of evidence by reducing the potential for bias (Katrak et al., 2004). Within healthcare, appraisal checklists have been found to improve the transparency of interventional studies, an important factor to ensure the review is open and honest about the methods used (Given, 2008). In this review, it is possible that local documents will be empirically derived from clinical expertise. As such, the critical appraisal will seek the credibility and authenticity of the opinions which underpin the text. It is also possible that the documents found will be formed using a mixture of expert opinion and primary and secondary research. Therefore, regardless of the methodological quality found, all documents will undergo data extraction and synthesis, as expert opinion is essential and uses a realistic approach to support the development of clinical guidelines (McArthur et al., 2017).

### Data synthesis

The extracted data will be gathered and synthesised using JBI SUMARI (Munn et al., 2018). Data synthesis is required to combine the conclusions found within the text. Synthesised conclusions enable a comparison of clinical practice, guidelines or opinion (Luo et al., 2018). The methodology for data synthesis can present a number of challenges. This includes transparency, consistency and a systematic approach to provide a credible review (Snilstveit et al., 2012). Therefore, data synthesis will be guided using a three-stage process of meta-aggregation (McArthur et al., 2017). To begin, the opinion statements extracted from the text of each document will be synthesised and concluded in order to generate a set of statements. Next, the statements will be assembled and categorised according to their similarity of meaning. Finally, each collection of statements will undergo meta-synthesis to create a new collection of findings which represent the meaning held within the data (Munn et al., 2018). If the levels of heterogeneity between the documents are high, gathering the extracted data into similar meanings may not be possible (McArthur et al., 2017). If this occurs, opinion statements and conclusions will be reported as a written account. The key conclusions drawn from the final synthesised data will be presented in a table and reported as a summary of findings. This detailed collection of synthesised findings aims to inform the evidence base, create new knowledge, provide recommendations and guide the future orientation of research for this topic area (Munn et al., 2018).

### Confidence in the cumulative evidence

The summary of findings table, which contains the main elements of the review, will be graded using the ConQual approach. ConQual aims to establish the quality and confidence of synthesis for qualitative reviews. A ConQual score will be provided for each synthesised finding and from the type of research which informs it (Munn et al., 2018).

## Acknowledgements

The reviewer would like to thank their supervisors for this collaboration and acknowledge the National Institute of Health Research and University of Plymouth for providing the opportunity and funding to complete the JBI systematic review module.

## Author contributions

AC is the main author of the protocol. RE and SB have made substantial contributions to the protocol search strategy, conducted a critical analysis of the protocol and drafted and approved the final version for submission to the Joanna Briggs Institute and journal publication. SJ proofread, amended and made substantial contributions to the protocol background. RE acts as the guarantor for this article.

## Conflict of interest

None declared.

## Ethics

Not required.

## Funding

The JBI systematic review training module was undertaken as part of the funded National Institute of Health Research integrated clinical academic award.

## Trial registration

PROSPERO 2019 (CRD42019138731).
